# Aluminum alters NMDA receptor 1A and 2A/B expression on neonatal hippocampal neurons in rats

**DOI:** 10.1186/1423-0127-18-81

**Published:** 2011-11-08

**Authors:** Chia-Yi Yuan, Guoo-Shyng Wang Hsu, Yih-Jing Lee

**Affiliations:** 1 Graduate Institute of Nutrition and Food Sciences, Fu-Jen Catholic University, 510 Chung-Cheng Road, Hsinchuang, New Taipei City 24205, Taiwan; 2 Department of Nutritional Science, Fu-Jen Catholic University, 510 Chung-Cheng Road, Hsinchuang, New Taipei City 24205, Taiwan; 3 School of Medicine, Fu-Jen Catholic University, 510 Chung-Cheng Road, Hsinchuang, New Taipei City 24205, Taiwan

**Keywords:** aluminum, neonates, primary hippocampal neuron, N-methyl-D-aspartate receptors, immunocytochemistry

## Abstract

**Background:**

High aluminum (Al) content in certain infant formula raises the concern of possible Al toxicity on brain development of neonates during their vulnerable period of growing. Results of in vivo study showed that Al content of brain tissues reached to 74 μM when oral intake up to 1110 μM, 10 times of that in the hi-Al infant formula.

**Methods:**

Utilizing a cultured neuron cells in vitro model, we have assessed Al influence on neuronal specific gene expression alteration by immunoblot and immunohistochemistry and neural proliferation rate changes by MTT assay.

**Results:**

Microscopic images showed that the neurite outgrowth of hippocampal neurons increased along with the Al dosages (37, 74 μM Al (AlCl_3_)). MTT results also indicated that Al increased neural cell viability. On the other hand, the immunocytochemistry staining suggested that the protein expressions of NMDAR 1A and NMDAR 2A/B decreased with the Al dosages (p < 0.05).

**Conclusion:**

Treated hippocampal neurons with 37 and 74 μM of Al for 14 days increased neural cell viability, but hampered NMDAR 1A and NMDAR 2A/B expressions. It was suggested that Al exposure might alter the development of hippocampal neurons in neonatal rats.

## Background

Aluminum (Al) is the second most abundant mineral in the soil, and it is also the major component of many legal food additives [1]. Al toxicities have been reported in renal disease patient with dialysis, due to high aluminum content in the dialysate and/or ingestion of Al-containing phosphate binder [2], resulting in microcytic hypochromic anemia, dialysis osteomalacia and dialysis encephalopathy [3]. The Al-content in the brain of person with Alzheimer's disease (AD) was reported to be higher than the age-matched non-AD elderly [4], although there are certain number of other reports disagreed with it [5,6]. Al over-loading has also been demonstrated in premature infants receiving intravenous fluid therapy [7]. These observations may imply that Al toxicity had a higher incidence in the population with kidney malfunction or immature kidney, such as nephropathy patients or in neonates. Although the absorption of Al in the gastrointestinal tract is less than 0.3%, and absorbed Al is mostly excreted through kidney in healthy individuals [8], the toxicity of dietary Al has raised concerns under certain patho-physiological, or even healthy conditions.

The nervous system, liver, and kidneys of human neonates are relatively immature during the first four weeks after birth [9], such that toxic substances may not be successfully detoxified by liver and excreted through kidneys during this period of development. Snell et al. (2001) reported that the blood-brain barrier of neonates has higher permeability than that in adults, increasing the probabilities of toxic substances diffusing into neural tissues [10]. It may affect the normal development of brain.

Since infant formula is the main food source for bottle-fed neonates, the Al content of infant formula deserves a greater concern. It has been reported that most skim milk or low fat milk contains less than 15 μM of elemental Al while some of the soy-based infant formulas contain up to 87 μM of Al [11]. Recent study also indicated that the mean Al content of ready to feed milk formulas ranged from 6.5 μM to 25.9 μM, and rehydrated milk formulas contained 12.3 μM to 23.3 μM [12]. In contrast, the Al concentration in human breast milk is only about 0.2-1.7 μM, 100 times lower than those found in infant formulas [11,13]. Furthermore, soy protein-based formulas in the USA have accounted for nearly 25% of the formula market [14]. Therefore, it is crucial to investigate whether the excessive Al in infant formula would accumulate in the brain tissues and disturb the brain development in neonates.

Several studies have shown that Al exposure during pregnancy affects maturation of motor neurons and learning capability in rats and rabbits [15,16]. High Al intake during gestation and lactation periods induces neurobehavioral defects, including foot slanting, reduction of thermal susceptibility and front-rear leg grasp ability in the pups [17]. These behavioral studies have suggested that Al may cause developmental change in nerve system, including hippocampus, cerebrum and cerebellum.

Since N-methyl-D-aspartate receptors (NMDARs) are widely expressed in the hippocampus and cortex [18] and the activation of NMDARs affects conduction between synapses and mediated synaptic plasticity in the central nervous system [19], therefore, they have been widely utilized as the biomarkers for development in these regions of the brain. There are several subunits of NMDARs, including NMDAR 1A, 2A and 2B, and the expression of these subunits are recognized to be developmentally regulated during postnatal period [20]. Stimulation of NMDARs could be associated with neural migration, regulation of axon and dendrite formation, synapse formation, cell death as well as selective degradation of synapses [21]. On the other hand, inhibition of NMDARs might cause defects in neural development [22]. These studies suggested that NMDAR activation may regulate neural development and differentiation. It is found that prenatal Al exposure impaired NMDARs neurotransmission in the cortex of pups [23].

There are certain studies using various level of Al to treat neural cells. *In vitro *studies suggested that Al (≤ 50 μM) promoted cerebellum granule cell, the smallest neurons, viability while high level of Al (≥ 100 μM) caused cell death [24]. Griffioen and his colleagues used 158.8 μM Al (aluminum maltolate) to treat human NT2 cells, a neural committed human teratocarcinoma cell line, which resulted in significant cell death after incubation for 24 hours [25]. At concentration of 180 to 630 μM, Al inhibited the expression of neural specific markers, microtubule-associated protein type 2 [26]. After 3-hour treatment of 500 μM (Al citrate), the neuronal viability was only 20% of the control [27]. One mM of Al suppressed the viability of cholinergic neurons [28], caused neural cell clustering and aggregation at days 4-6 of incubation, and cell death at days 8-12 [29].

The purpose of this study was to investigate the effects of Al at physiologic levels (attainable from dietary source) on hippocampal neural development during postnatal period by an *in vitro *model, using the biomarker NMDARs expression. The hypothesis was that elevated Al levels up to 37 and 74 μM would decrease neural cell viability and NMDAR 1A, 2A/B expressions. The findings of this study may provide valuable information to establish recommendations for selecting infant formula.

## Methods

### Primary hippocampal neuron culture

Sprague-Dawley (SD) rats (250 g~300 g) were kept under a 12 hours dark/12 hours light cycle, at 20-22°C. Rodent laboratory chow (Purina Lab. Chow 5001, St. Louis, MO) and water were available *ad libitum*. After mating, the pregnant females were individually housed in plastic cages. The animal experimental procedure was compliant to the guidelines of the National Science Council in Taiwan and approved by the IACUC (Institutional Animal Care and Use Committee) of the Fu-Jen University.

Although there is no direct evidence to prove that one-day of cell culture equivalent to one-day growth of neonates, a primary culture of hippocampal neurons from embryonic day 19 (E19) prenatal rat embryo was adopted for 6 days to mimic the neurons in postnatal day 3 (PND3) neonates in vivo [2,30].

The uterus of SD rats at E19 was removed under anesthesia. Fetus was separated and the brains were dissected and placed into 1 M HBSS (Gibco 14170-013). Under a stereo microscope (Motic^® ^Microscope), the hippocampus was removed from each pups. The hippocampal cells were dissociated by using 0.25% trypsin (Gibco 15050-065). Cells were taken up in NEUROBASAL™-A Medium (Gibco 21103-049) complex, containing serum-free B27 supplement, GlutaMax™-supplement, 25 μM glutamate, 25 mM β-mercaptoedianol, penicillin and streptomycin [31], which was designed for neural cell culture containing the inhibitors of glial cells [32]. In addition, nerve growth factor (100 ng/ml, NGF-7S, Sigma N0513) was added into the medium. The hippocampal neural cells were plated onto poly-D-lysine (Sigma P-9011) and laminin (Sigma L-2020)-coated cover slips [33] inside of a 24-well (TPP^® ^92024, 9.5 × 10^4^/per well). The isolated neurons were allowed to grow at 37°C in 5% CO_2_/95% air and the medium was changed every 3 days. In general, one pregnant female rat had 10-12 fetus and about 5-6 plates of 24-well plates of hippocampal neurons could be isolated.

### Treatments with aluminum

0, 37 and 74 μM of aluminum chloride (AlCl_3_, Merck 801081) was added respectively into the culture medium on day 6 (PND3), and the cells were treated for 7 or 14 days, to mimic the period of birth to weaning in vivo study. AlCl_3 _solution prepared in sterile deionized distilled water, the pH value was adjusted to 7.4 and mixed before used (Al content ≦ 5 ppb). Culture medium with or without fresh-made Al was changed every 3 days. The Al dosages added in the culture medium were based on previous pilot study in our lab. The Al concentration in the whole brain of neonatal rats (PND 17) were 0.79 ± 0.24 mg/L, 0.83 ± 0.18 mg/L and 1.44 ± 0.72 mg/L when neonates fed artificial rat's milk with various Al contents (0, 1 and 2 mM AlCl_3 _respectively from PND 3 to 17). Therefore, physiological levels of Al in brain tissues are between 0 and 2 mg/L (0-74 μM).

### Cell morphology and viability

The cellular morphological images of hippocampal neurons were obtained by a inverted microscope and a digital image system (LEICA DMIL and DFC350FX). The viability (cell counting) of neurons was examined by the 3-(4,5-dimethylthiazo)-2,5-diphenyltetrazolium bromide (MTT) test. Fresh-made MTT solution, 5 mg/ml in Dulbecco's Modified Eagle's Medium (DMEM) was added to the medium. The cells were incubated at 37°C, with 5% CO_2 _for two hours. The MTT solution was removed after incubation and 500 μl DMSO (dimethyl sulfoxide) was added to each well. Optical density was measured at 550 nm with ELISA reader (μQuant, Bio-TEK instrument). The results are presented by using the mean of control group as 100%, and the cell viability in each experimental group is expressed as percentage of the control group.

### Immunocytochemistry

Dual-Immunofluorescence staining was conducted from using a published method [33]. Briefly, cultured neural cells were fixed in 4% paraformaldehyde in PBS and washed with PBS. PBS with 5% FBS was added to the cells as blocking buffer. After removing the solution, the cultured cells were incubated with primary antibodies (mouse anti-NMDAR 1A monoclonal antibody (Chemicon international MAB363) and rabbit anti-NMDAR 2A/B polyclonal antibody (Chemicon international AB1548)) at 4°C overnight. After incubation with secondary antibodies, including donkey anti-mouse IgG (Alex Fluor 488) and donkey anti-rabbit IgG (Alex Fluor 555), cell nuclei were stained by DAPI (Santa Cruz Biotechnology, sc-3598). The cells were then mounted (DakoCytomation), and fluorescent images were captured by a fluorescent microscope (LEICA DM2500) and a digital image system (Photometrics Cool SNAP™ EZ). The fluorescent signal levels of NMDAR 1A, 2A/B were quantified, using the chemoluminescence/fluorescence spectrometer (Perkin Elmer Precisely Wallac Victor3™ 1420 multi-lable counter). The fluorescent content was calibrated by the cell number per well and the protein expression was expressed as the amount of specific protein in each cell. The protein level in each experimental group was expressed as percentage of the control.

### Statistical analysis

Data was analyzed using a SAS package (Statistical Analysis System, version 9.2). All values were expressed as mean ± standard deviation. ANOVA was used to analyze the differences among the variable followed by Scheffe's multiple comparisons. The significance of difference was determined as p < 0.05.

## Results

**Figure 1:**
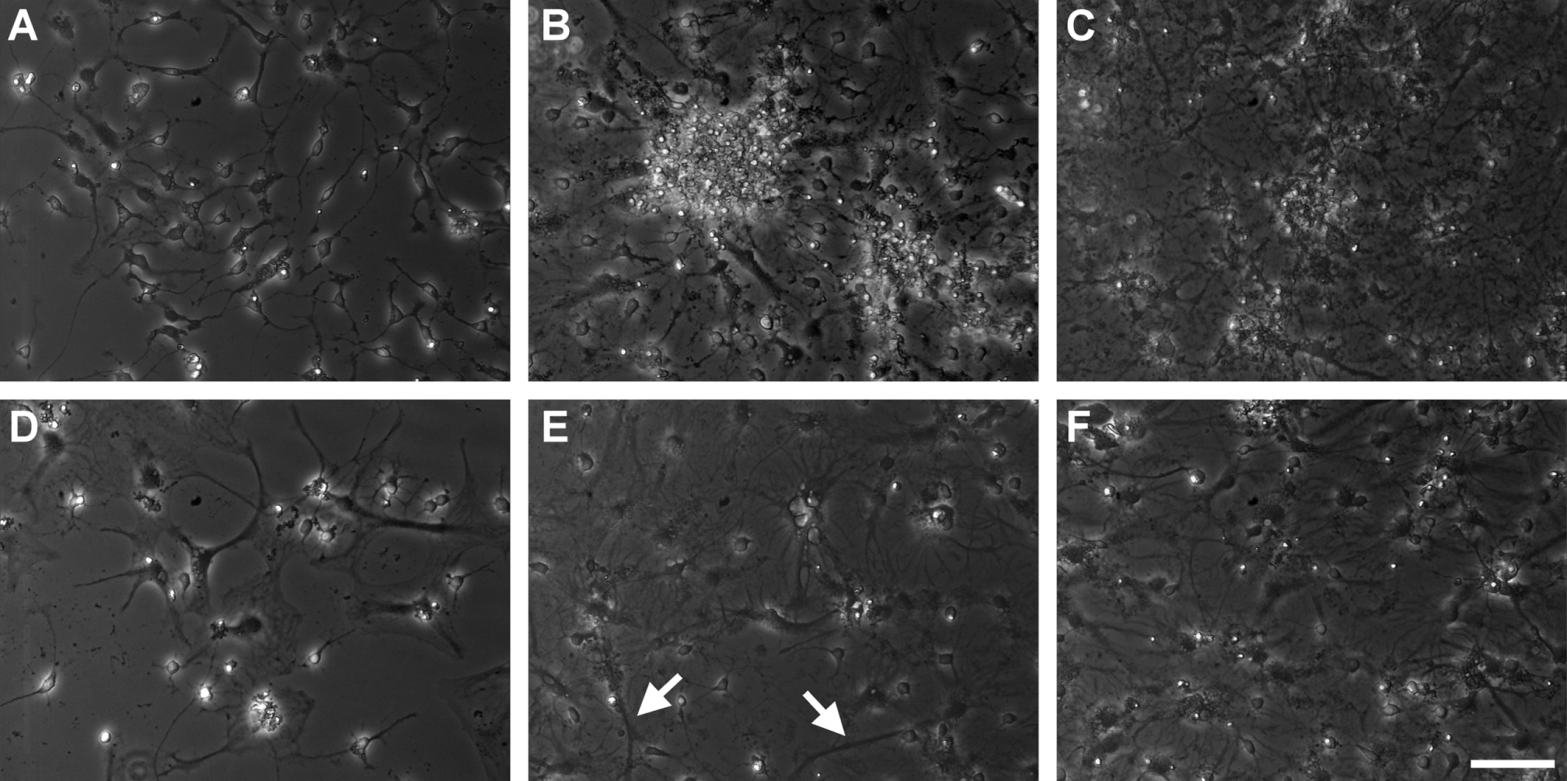
**Microscopic images of cultured hippocampal neurons**. (A), (B), and (C) are neurons cultured with 0, 37 and 74 μM Al respectively for 7 days (PND 10). (D), (E) and (F) are neurons cultured with 0, 37 and 74 μM Al respectively for 14 days (PND17). White arrows label the neurite outgrowth and white line shows the scale bar expressed as 50 μm.

Figure 1 showed the microscopic images of cultured hippocampal neurons (200 ×). Based on morphological observation, Figure 1b (37 μM, 7 days) and Figure 1c (74 μM, 7 days) seemed to have more neural cells than the control group (Figure 1a). Figure 1e (37 μM, 14 days) and 1f (74 μM, 14 days) appeared to have more neural cell, compared to the control group (Figure 1d). More interconnections between aggregates of hippocampal neurons were also observed in 74 μM Al groups cultured for 7-days (Figure 1c) and 14-days (Figure 1f), compared to the control groups accordingly (Figure 1a, d). In addition, increased neurite outgrowth (white arrows) at PND 17, as illustrated in Figure 1e were also observed in hippocampal neurons of Al treated cells.

Quantitative results of cell viability of hippocampal neurons were shown as Table 1. The result of statistical analysis indicated that the neural cell viability would increase along with the Al exposure dosage. The cell numbers of three groups (0, 37 and 74 μM Al) were (2.1 ± 1.3) × 10^4^, (4.8 ± 1.5) × 10^4 ^and (6.9 ± 2.5) × 10^4 ^respectively (p < 0.05). The neural cell viability would also increase along with the Al exposure time, and the cell numbers of two groups (7 and 14 day) were (3.9 ± 1.8) × 10^4 ^and (6.3 ± 2.8) × 10^4 ^respectively (p < 0.05). Neuronal cell viability significantly increased by 3.08 and 4.34 times compared to the control group ((1.2 ± 0.3) × 10^4^) in cells treated with 37 and 74 μM Al treated for 7 days ((3.8 ± 0.8) × 10^4^, (5.4 ± 1.2) × 10^4^, p < 0.05). Compared to the control group((3.0 ± 1.2) × 10^4^), neuronal cell viability significantly increased by 1.94 and 2.78 times in cells treated with 37 and 74 μM Al for 14 days ((5.8 ± 1.5) × 10^4^, (8.4 ± 2.6) × 10^4^, p < 0.05).

**Table 1:** The cell viability of cultured hippocampal neurons by MTT-test ^1,2,3^

Time (day)	Al level (×10^4 ^μM)			Time effect
		
	0	37	74	
7	1.2 ± 0.3 ^CY^	3.8 ± 0.8 ^BY^	5.4 ± 1.2 ^AY^	3.9 ± 1.8 ^Y^
14	3.0 ± 1.2 ^BX^	5.8 ± 1.5 ^BX^	8.4 ± 2.6 ^AX^	6.3 ± 2.8 ^X^

**Dosage effect**	2.1 ± 1.3 ^C^	4.8 ± 1.5 ^B^	6.9 ± 2.5 ^A^	

^1. ^All values are Mean ± SD (cell numbers).

^2. ^Values in the same row with different ^A, B, C ^superscripts are significantly different at α = 0.05 level.

^3. ^Values in the same column with different ^X, Y ^superscripts are significantly different at α = 0.05 level.

**Figure 2:**
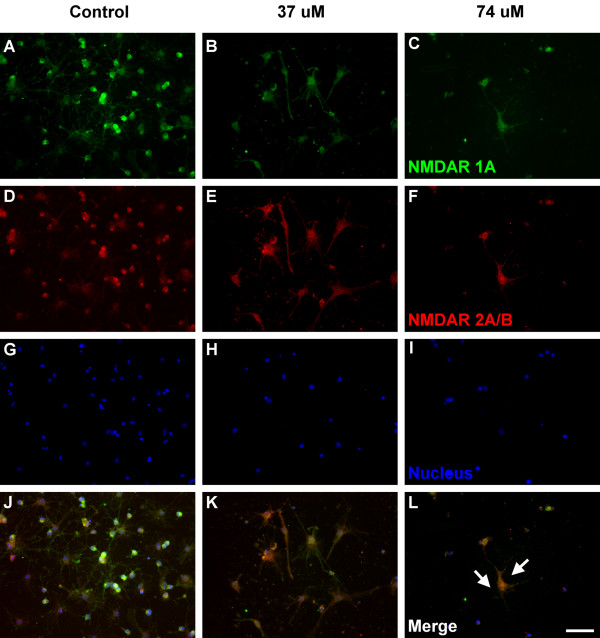
**Fluorescence immunocytochemical images of the cultured hippocampal neurons**. Green (A, B, C), red (D, E, F) and blue (G, H, I) fluorescence represent NMDAR 1A, NMDAR 2A/B and the nucleus of the cells, respectively. (J), (K) and (L) are neurons cultured with 0, 37 and 74 μM Al for 7 days (PND 10). White arrows label the cell disruption and white line shows the scale bar expressed as 50 μm.

**Figure 3:**
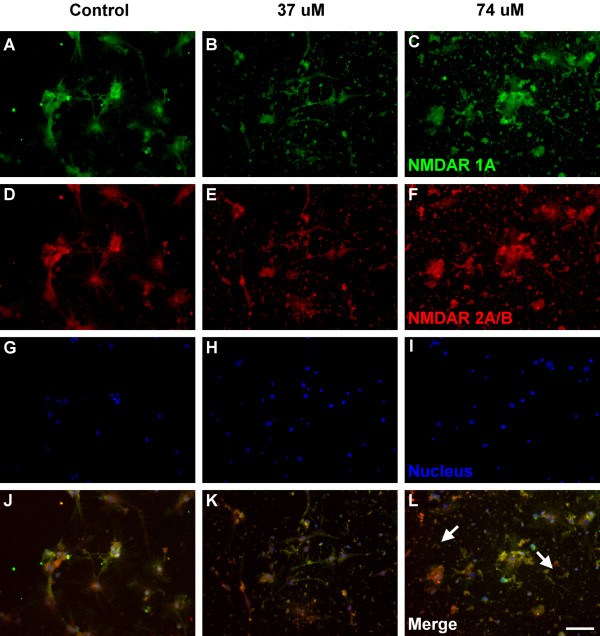
**Fluorescence immunocytochemical images of the cultured hippocampal neurons**. Green (A, B, C), red (D, E, F) and blue (G, H, I) fluorescence represent NMDAR 1A, NMDAR 2A/B and the nucleus of the cells, respectively. (J), (K) and (L) are neurons cultured with 0, 37 and 74 μM Al for 14 days (PND 17). White arrows label the cell debris and white line shows the scale bar expressed as 50 μm.

The immunocytochemical analysis for the expression of NMDAR 1A (green color) and NMDAR 2A/B (red color) were shown as Figure 2 and 3. NMDAR 1A and NMDAR 2A/B were both detected on the entire neuron, including cell membrane and cytoplasm (Figure 2j). Based on morphological observation, cells in Figure 2k (37 μM, 7 days) and 2l (74 μM, 7 days) seem to have less NMDARs, compared to the control group (Figure 2j). Cell disruption (white arrows) was observed in neurons treated with 74 μM Al for 7 days (Figure 2l) and cell debris (white arrows) was observed in neurons treated with 74 μM Al for 14 days (Figure 3l). These findings suggested that Al might urge the NMDARs neurons to cell disruption, debris and death.

**Figure 4:**
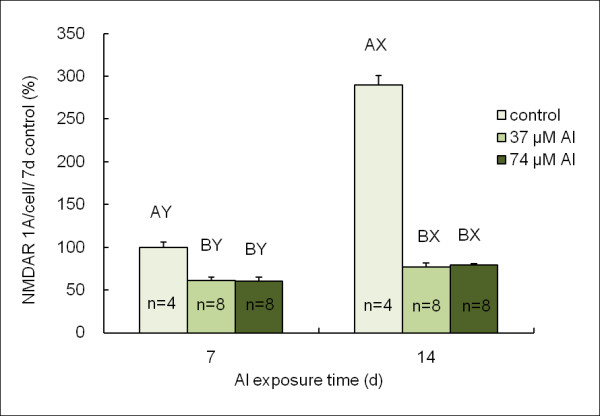
**Quantitative analysis of NMDAR 1A expression**. There are significant difference (P < 0.05) of values with different superscripts A, B, at the neurons which were cultured with 0, 37 and 74 μM Al at the same time point. There are significant difference (P < 0.05) of values with different superscripts X, Y, at the 7^th ^day (PND 10) and the 14^th ^day (PND 17) at the same Al level. NMDAR 1A expression in 7-day control was considered as 100%.

Quantitative analysis of NMDAR 1A expression was shown in Figure 4. The NMDAR 1A expressions per cell in Al treated groups (both 37 and 74 μM) were lower than that in the control group in accordance with time (p < 0.05). Cells treated with 37 and 74 μM Al for 7 days showed decreased NMDAR 1A expressions by 39% and 40%, respectively, compared to the control group (p < 0.05). The NMDAR 1A expressions in cells treated with 37 and 74 μM Al for 14 days both dropped 73% compared to the control group (p < 0.05).

**Figure 5:**
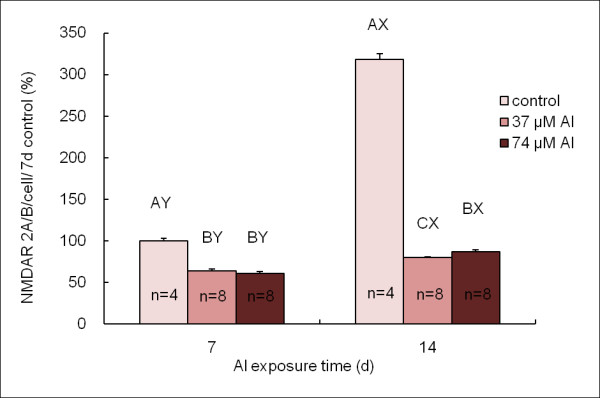
**Quantitative analysis of NMDAR 2A/B expression**. There are significant difference (P < 0.05) of values with different superscripts A, B, C, at the neurons which were cultured with 0, 37 and 74 μM Al at the same time point. There are significant difference (P < 0.05) of values with different superscripts X, Y, at the 7^th ^day (PND 10) and the 14^th ^day (PND 17) at the same Al level. NMDAR 2/AB expression in 7-day control was considered as 100%.

Similar result was observed in the NMDAR 2A/B expressions (Figure 5). The expressions of NMDAR 2A/B per cell decreased in Al treated groups for both 7 days and 14 days (Figure 5, p < 0.05). Compared to the control group, NMDAR 2A/B expressions decreased 36% and 39% in the cells treated with 37 and 74 μM Al for 7 days respectively (p < 0.05), and 75% and 73% in the cells treated with 37 and 74 μM Al exposure for 14 days respectively (p < 0.05).

## Discussion

In this study, we found that treating culturing neurons with 37 and 74 μM of Al increased neurite outgrowth, an index of neural differentiation, in the developing neuron. Evidences had pointed out that the events of sprouting and neurite outgrowth were associated with an increased tyrosine-tubulin (Tyr-Tub) expression which enhanced the neuronal plasticity. Meanwhile, extra cellular matrix molecules and cell adhesion molecules could also promote neurite outgrowth [34]. One study had indicated that 10-20 μM Al (aluminum lactate) promotes neuronal sprouting and neurite outgrowth are associated with an increased tyrosine-tubulin (Tyr-Tub) expression in a mouse neuroblastoma cell line after 48 and 72 h of Al exposure [35]. The result of Al accelerating neuronal sprouting and neurite outgrowth was similar to our study. Also as a trivalent element, neurons seeded on gallium nitride (GaN) were able to form an extensive neurite network and neurite outgrowth [36]. However, there is still a few evidence showing possible mechanism of Al affecting neurite outgrowth directly. Further investigation is needed for elucidating the possible pathways Al may be involved in neurite outgrowth.

As mentioned earlier, many researches had proved that exposure to high level of Al (150 μM-1 mM) could decrease the neural cell numbers and cause cell death. However, few study concerned about the effect of physiological concentrations of Al (< 100 μM) on neural cells. There was a study reported that low levels of Al (≤ 50 μM) promoted cerebellum neural cell viability while high levels of Al (≥ 100 μM) caused cell death [24]. Our results indicated that cell viability was still enhanced in developing or neonatal neurons even with Al content up to 74 μM. Nonetheless, physiological levels of Al still could cause some subtly negative influences to the neural cell. Low levels (100 nM to 2 *μ*M Al_2 _(SO_4_)_3_) of Al appeared to induce a stress-responsive, pro-inflammatory and pro-apoptotic gene expression program that may initiate, enhance and/or accelerate neural cell demise, both neuronal- and glial-specific genetic output in both isolated human brain cell nuclei [37] and cultured human neural cells [38].

In the limbic system of mammals, the hippocampus is crucial for processes of learning and long-term memory. Since NMDARs play an important role on neural cell growth and differentiation in hippocampus, expression of NMDARs in hippocampus has been implicated as important indicators of both learning and neural development [39]. Several studies indicated that there is NMDAR 1A/2B subunits combination in immature brain and NMDAR 1A/2A subunits combination in mature brain [40,41]. The distribution and variation of NMDARs subunits in the developmental process might constitute the functional divergence in brain nerve system. In present study, protein expressions of NMDAR 1A and NMDAR 2A/B decreased while increasing dosages of Al in culture medium (37 and 74 μM). It was also suggested that overexposure of Al caused neural excitability and decreased cell viability, possibly mediated by NMDARs [23,42]. Our results was also confirmed with report of Exley et al. [43] that Al could induce toxic effects on neurons even at physiological concentrations. Other report indicated that the threshold concentration of Al without inducing negative effects on NMDA is currently set at < 10 μM [42]. This study concluded that exposure to physiological concentrations (0-74 μM) of Al might cause the developmental change of hippocampus in neonatal rats.

The results of fluorescence immunocytochemical images suggested that Al could hamper the hippocampal neurons that express NMDARs proteins, although the neural cell viability of the hippocampus was enhanced by 37 and 74 μM of Al. Walton's study suggested that Al induced the hippocampal lesion of aged rats and AD people, looked like cell disruption, consisting of dysfunctional Al-rich microtubule-depleted pyramidal cells with damaged neurites and synapse loss [44]. Therefore, proliferated cells might express functional proteins other than NMDARs. Our results also confirmed that Al exposure caused decrement of NMDARs expression during developmental period of neonatal rats.

The possible mechanisms of Al toxicity on neurons in brain was also an important issue. Al might block ion uptake of Ca^2+^, Fe^2+^, Mg^2+^, and Na^+ ^that affected neuronal metabolism [45]. Al might induce free radical mediated lipid peroxidation, increment of oxidative stress and cell damage in glioma and neuroblastoma [46]. Physiologically relevant amounts of iron and aluminum are capable of inducing Fenton chemistry-triggered gene expression programs that may support downstream pathogenic responses and brain cell dysfunction [47]. Al might also stimulate G-proteins associated with second messenger system in vivo [48]. Kim showed that prenatal exposure to Al altered neuronal nitric oxide synthase expression in the frontal cortex of rat offspring [49]. It was suggested that Al had high affinity to nucleic acid, allowing accumulation of Al in chromosome of hippocampal pyramidal neurons and neuron death [50]. Al also affects enzymes that maintain brain function, such as cholinesterase [51] and superoxide dismutase [52]. Al may inhibit Ca^2+ ^channel; thus increase permeability and fluidity of neural cell membrane [53]. In our study, we suggested that Al might induce neurotoxicity by hampering protein expressions of NMDAR 1A and NMDAR 2A/B.

**Figure 6:**
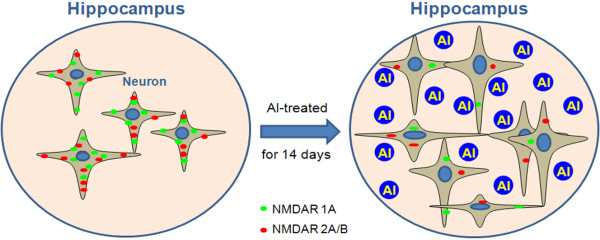
**This illustration demonstrates the possible mechanism involved in Al effect on neonatal hippocampal neurons**. Green and red fluorescence represent NMDAR 1A and NMDAR 2A/B in the hippocampal neural cells, respectively.

The final shematic conclusions of the present study included four points (Figure 6). Al enhances neonatal hippocampal neurite outgrowth and neural cell viability. Al hampers the expression of NMDAR 1A and 2A/B of the hippocampal neurons.

## Conclusions

In conclusion, treating hippocampal neurons with 37 and 74 μM of Al for 14 days enhanced neural cell viability, but down-regulated the expressions of NMDAR 1A and NMDAR 2A/B. It was suggested that Al exposure might alter the development of hippocampal neurons in neonatal rats. The mechanisms by which Al increases neural cell viability and decreases NMDARs expressions will need further investigation.

## List of abbreviations

Al: aluminum; NMDAR: N-methyl-D-aspartate receptor; E 19: embryonic day 19; PND: postnatal day; MTT: 3-(4,5-dimethylthiazo)-2,5-diphenyltetrazolium bromide.

## Competing interests

The authors declare that they have no competing interests.

## Authors' contributions

CYY designed the experimental protocol, performed the experiments and drafted the manuscript. GSWH contributed to the study concept, research design, data interpretation and manuscript revision. YJL contributed to the study concept, research design, data interpretation, manuscript revision and did the image technical appraisement. All authors read and approved the final version of the manuscript.
